# Supplementary material for: Functional Study of PgHDZ01 Gene Involved in the Regulation of Ginsenoside Biosynthesis in Panax ginseng
Source: Plants (Basel). 2025 Nov 21;14(23):3562. doi: 10.3390/plants14233562 (PMC12693816; doi:10.3390/plants14233562)
Supplement: Supplementary file 1 [file plants-14-03562-s001.zip › Supplemental Figure.S1-S4.pdf]

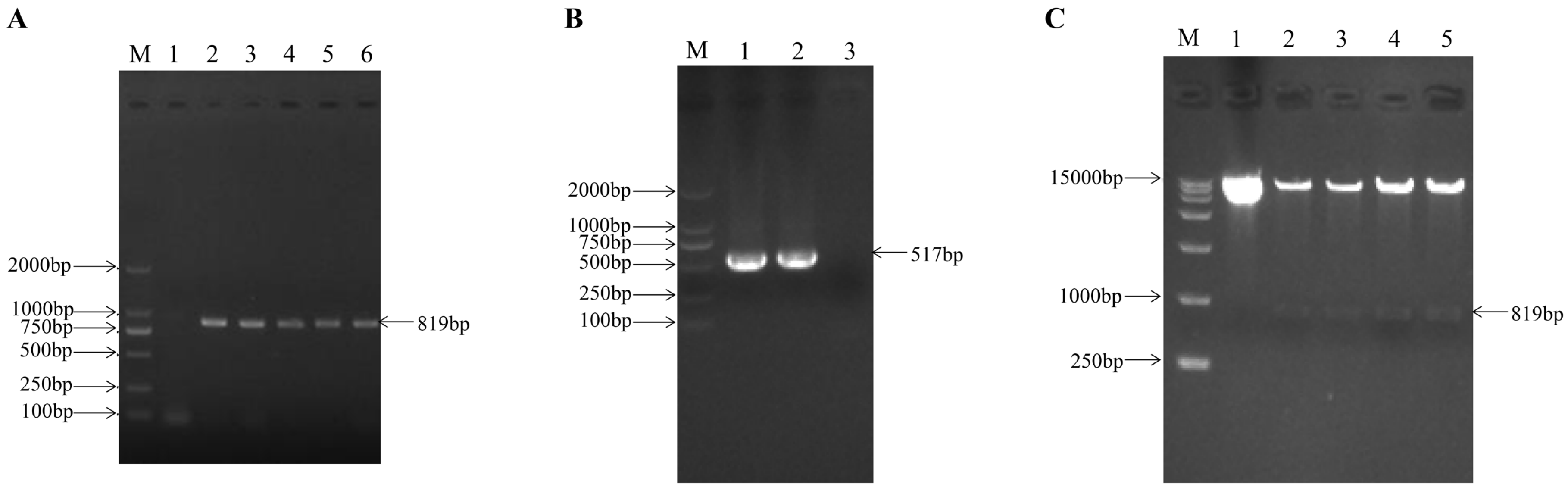

Supplemental Figure S1. (A) The PCR result of *PgHDZ01* gene. M, DL2000; Line 1, Blank; Lines 2-6, PCR result. (B) The PCR result of *PgHDZ01*-VIGS gene. M, DL2000; Line 3, Blank; Lines 1-2, PCR result. (C) The *Xma*I digest result of recombinant vector of pCAMBIA3301: *PgHDZ01*. M, DL15,000; Line 1, recombinant vector; Lines 2-5, digest result of recombinant vector.

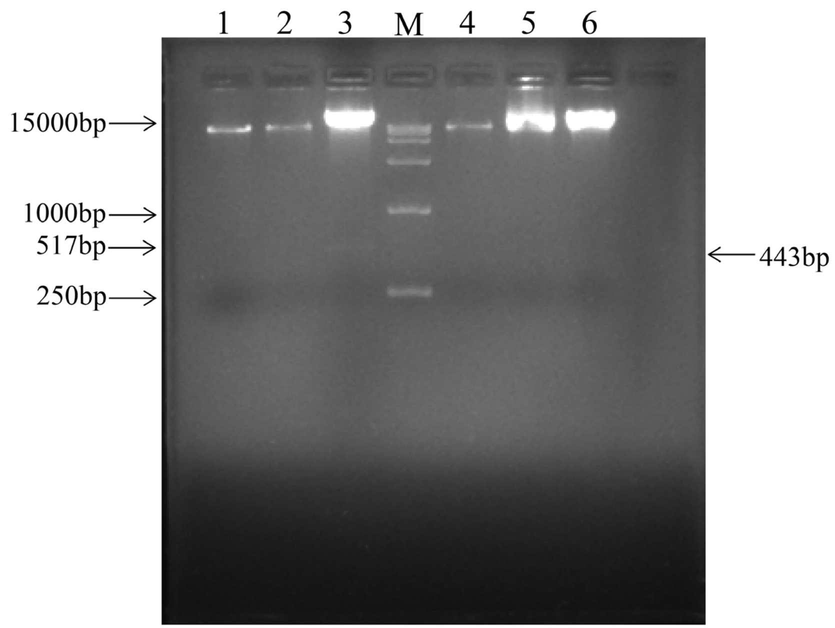

Supplemental Figure S2. Construction of the VIGS gene silencing vector. The *Eco*RI and *Kpn*I double digest results of recombinant vectors pGM-T: *PgHDZ01* and pGM-T: *PgPDS.* M, DL15000; Lines 3 and 6, double digest result; Lines 1 and4, *Eco*RI digest result; Lines 2 and 5, *Kpn*I digest result.

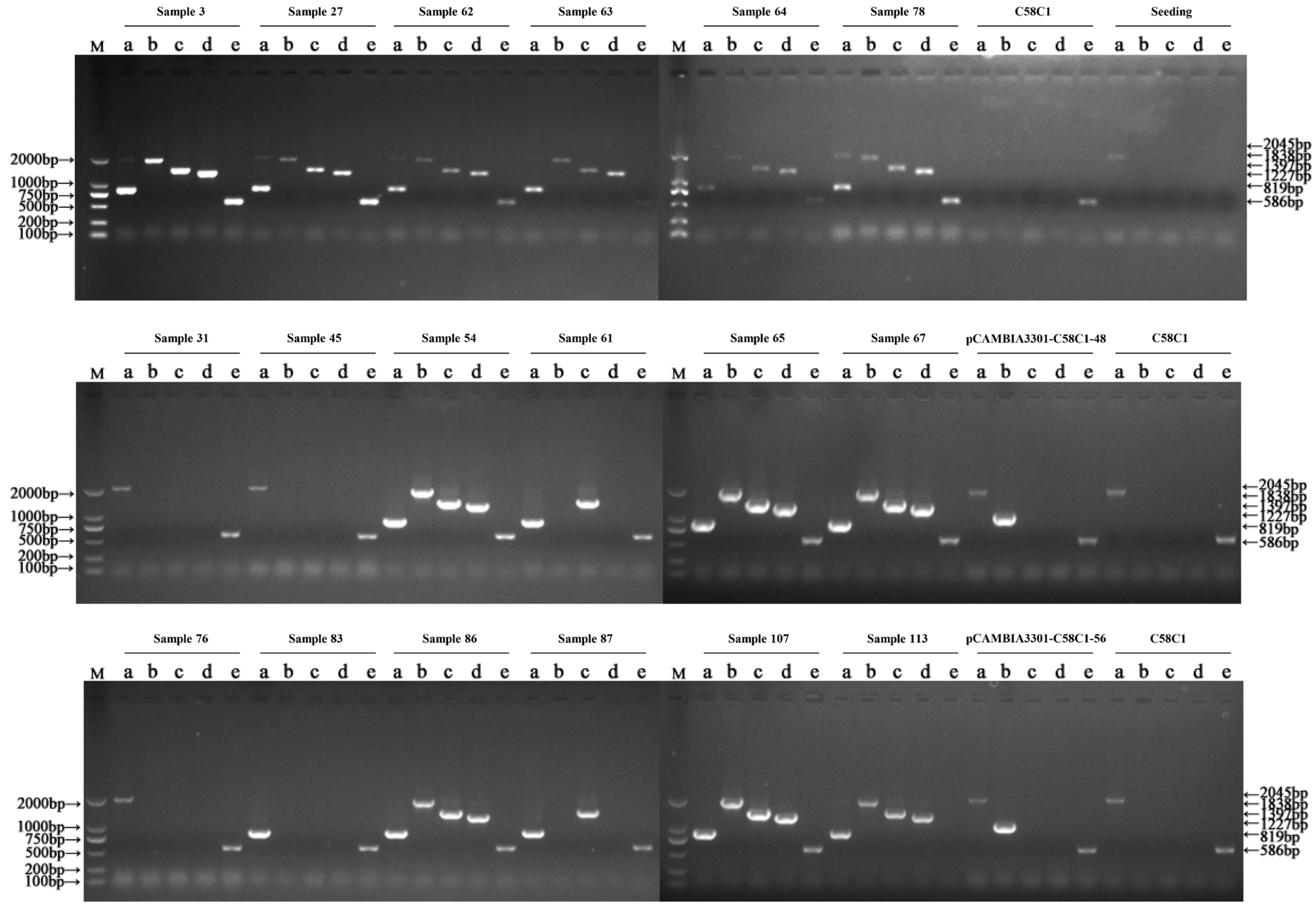

Supplemental Figure S3. The PCR detection of ginseng hairy roots that overexpressed *PgHDZ01*. M, DL2000; a, *PgHDZ01* gene; b, partial vector with *PgHDZ01* gene; c, upstream partial vector with *PgHDZ01* gene; d, downstream partial vector with *PgHDZ01* gene; e: *RolC* gene.

A.

B.

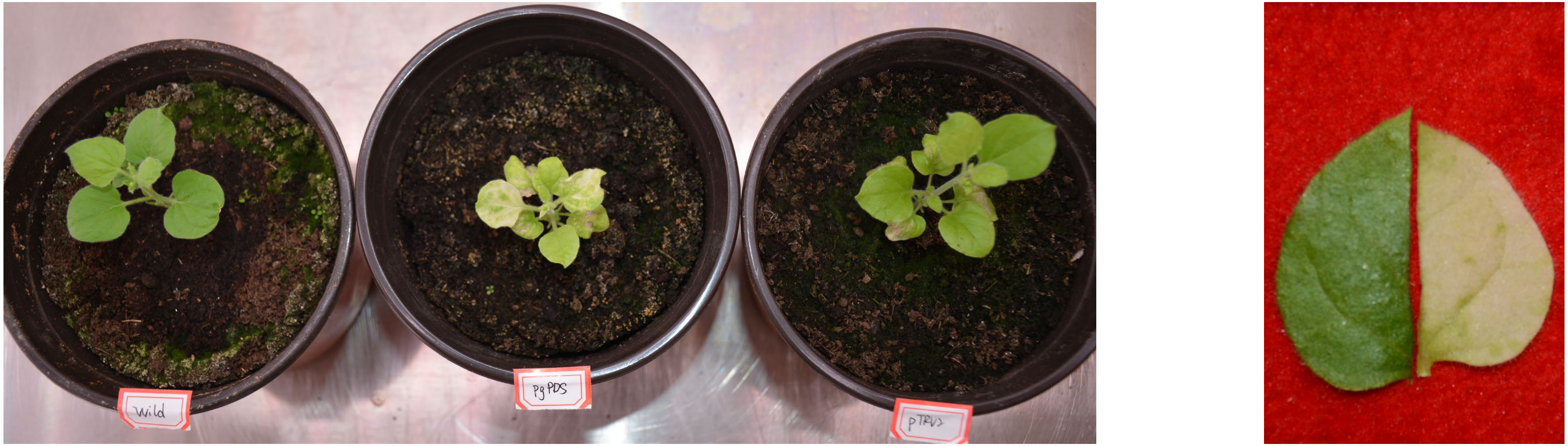

Supplemental Figure S4. VIGS-mediated silencing of *PgPDS* gene in *Nicotiana benthamiana*. (A) Left: wild plant; Center: pTRV2: *PgPDS* silenced plant; Right: pTRV2: 00 silenced plant. (B) Comparison of wild tobacco leaf and silenced *PgPDS* gene leaf. Left: wild leaf; Right: pTRV2: *PgPDS* silenced leaf
