# Supplementary material for: Functional Study of PgHDZ01 Gene Involved in the Regulation of Ginsenoside Biosynthesis in Panax ginseng
Source: Plants (Basel). 2025 Nov 21;14(23):3562. doi: 10.3390/plants14233562 (PMC12693816; doi:10.3390/plants14233562)
Supplement: Supplementary file 1 [file plants-14-03562-s001.zip › Supplemental Table S1.pdf]

Supplemental Table S1. *PgHD-Zip* genes that significant related to ginsenoside content.

Gene		Rg1	Re	Rf	Rb1	Rg2	Rc	Rb2	Rb3	Rd	Total saponin
PgHDZ01	Correlation Coefficient	-0.091	-0.162	**-.401****	**-.333***	-0.284	**-.453****	-0.102	0.047	**-.410****	**-.489****
	Sig. (2-tailed)	0.564	0.304	0.009	0.031	0.068	0.003	0.521	0.768	0.007	0.001
PgHDZ02	Correlation Coefficient	-0.155	0.043	0.009	**-.306***	0.122	-0.02	-0.215	0.056	-0.223	-0.228
	Sig. (2-tailed)	0.326	0.786	0.953	0.048	0.443	0.902	0.171	0.726	0.156	0.146
PgHDZ06-02	Correlation Coefficient	0.098	-0.169	0.209	-0.163	0.107	0.045	0.264	**.305***	0.086	0.001
	Sig. (2-tailed)	0.537	0.285	0.183	0.303	0.5	0.776	0.091	0.05	0.586	0.997
PgHDZ07	Correlation Coefficient	-0.009	**-.316***	0.192	-0.061	-0.142	0.189	-0.214	0.009	-0.217	-0.066
	Sig. (2-tailed)	0.957	0.042	0.223	0.703	0.37	0.23	0.173	0.954	0.167	0.679
PgHDZ09	Correlation Coefficient	0.285	0.021	-0.034	**.307***	0.085	-0.097	0.071	-0.051	0.075	0.165
	Sig. (2-tailed)	0.067	0.893	0.829	0.048	0.591	0.54	0.654	0.746	0.637	0.298
PgHDZ10-01	Correlation Coefficient	-0.051	0.101	-0.2	0.19	**-.309***	-0.086	-0.202	-0.116	-0.101	-0.037
	Sig. (2-tailed)	0.75	0.523	0.203	0.227	0.047	0.59	0.201	0.466	0.526	0.815
PgHDZ10-02	Correlation Coefficient	-0.061	**.307***	0.095	0.19	0.09	**.361***	0.162	-0.188	0.164	0.239
	Sig. (2-tailed)	0.703	0.048	0.551	0.229	0.569	0.019	0.304	0.233	0.298	0.127
PgHDZ13-01	Correlation Coefficient	**.307***	-0.122	0.304	0.237	0.164	-0.143	-0.043	-0.189	0.248	0.218
	Sig. (2-tailed)	0.048	0.443	0.05	0.13	0.299	0.365	0.786	0.231	0.113	0.166
PgHDZ13-03	Correlation Coefficient	-0.081	**.425****	0.146	0.015	0.166	-0.111	0.092	-0.126	-0.098	-0.047
	Sig. (2-tailed)	0.611	0.005	0.356	0.926	0.293	0.484	0.563	0.426	0.538	0.768
PgHDZ14	Correlation Coefficient	-0.184	**.473****	0.076	**.325***	0.204	0.121	-0.092	-0.037	0.22	**.311***
	Sig. (2-tailed)	0.243	0.002	0.631	0.036	0.195	0.445	0.562	0.816	0.162	0.045
PgHDZ15-03	Correlation Coefficient	**.397****	0.032	0.241	**.511****	0.004	0.164	0.088	0.018	**.481****	**.524****
	Sig. (2-tailed)	0.009	0.841	0.125	0.001	0.982	0.3	0.579	0.91	0.001	0.001
PgHDZ17-01	Correlation Coefficient	0.149	0.169	-0.109	**.389***	-0.169	0.01	0.121	-0.127	0.214	0.251
	Sig. (2-tailed)	0.348	0.284	0.494	0.011	0.284	0.95	0.445	0.421	0.174	0.109
PgHDZ17-02	Correlation Coefficient	0.089	-0.007	-0.069	**-.329***	-0.122	-0.116	-0.096	-0.1	**-.347***	-0.299
	Sig. (2-tailed)	0.576	0.965	0.663	0.033	0.443	0.465	0.545	0.529	0.024	0.055
PgHDZ17-03	Correlation Coefficient	**.344***	0.05	0.128	0.243	0.047	0.082	0.05	-0.275	0.246	0.281
	Sig. (2-tailed)	0.025	0.754	0.421	0.121	0.769	0.607	0.755	0.078	0.117	0.071
PgHDZ18-01	Correlation Coefficient	0.162	-0.304	-0.271	-0.106	**-.404****	-0.252	0.006	-0.039	-0.015	-0.179
	Sig. (2-tailed)	0.306	0.051	0.082	0.502	0.008	0.107	0.972	0.804	0.923	0.258
PgHDZ18-04	Correlation Coefficient	-0.126	-0.119	**-.315***	-0.212	-0.107	-0.121	-0.135	-0.299	0.088	-0.134
	Sig. (2-tailed)	0.425	0.452	0.042	0.178	0.501	0.447	0.393	0.054	0.58	0.397
PgHDZ18-05	Correlation Coefficient	0.12	0.179	**.355***	**.386***	**.319***	0.173	-0.099	-0.06	**.350***	**.422****
	Sig. (2-tailed)	0.45	0.257	0.021	0.011	0.04	0.274	0.533	0.704	0.023	0.005
PgHDZ18-06	Correlation Coefficient	-0.029	0.076	**-.321***	**-.322***	-0.176	-0.2	-0.152	-0.277	**-.369***	**-.350***
	Sig. (2-tailed)	0.858	0.634	0.038	0.038	0.265	0.205	0.335	0.075	0.016	0.023
PgHDZ18-07	Correlation Coefficient	-0.193	-0.074	**-.404****	-0.123	-0.227	-0.036	-0.184	**-.320***	0.129	-0.089
	Sig. (2-tailed)	0.221	0.641	0.008	0.436	0.149	0.82	0.244	0.039	0.415	0.577
PgHDZ18-10	Correlation Coefficient	0.25	-0.121	-0.067	0.26	-0.116	-0.002	-0.078	-0.127	**.378***	0.258
	Sig. (2-tailed)	0.111	0.446	0.676	0.097	0.463	0.992	0.623	0.421	0.014	0.099
PgHDZ18-11	Correlation Coefficient	0.249	-0.094	0.023	0.219	-0.033	-0.082	-0.047	-0.165	**.356***	0.216
	Sig. (2-tailed)	0.112	0.554	0.885	0.163	0.834	0.607	0.768	0.297	0.02	0.169
PgHDZ18-14	Correlation Coefficient	0.137	0.151	**.307***	**.428****	0.258	0.254	-0.183	-0.124	**.319***	**.461****
	Sig. (2-tailed)	0.388	0.339	0.048	0.005	0.099	0.104	0.247	0.433	0.04	0.002
PgHDZ18-21	Correlation Coefficient	0.114	-0.289	**-.327***	-0.097	**-.441****	-0.213	-0.034	-0.077	0.006	-0.177
	Sig. (2-tailed)	0.471	0.064	0.035	0.541	0.004	0.175	0.832	0.63	0.972	0.262
PgHDZ18-23	Correlation Coefficient	**.361***	0.089	0.192	**.307***	0.101	0.067	-0.272	**-.309***	0.232	**.315***
	Sig. (2-tailed)	0.019	0.574	0.224	0.048	0.523	0.671	0.082	0.046	0.139	0.042
PgHDZ18-28	Correlation Coefficient	-0.006	0.127	**-.339***	-0.275	-0.167	-0.224	-0.243	**-.339***	**-.398****	**-.388***
	Sig. (2-tailed)	0.97	0.422	0.028	0.078	0.29	0.154	0.121	0.028	0.009	0.011
PgHDZ18-29	Correlation Coefficient	**.321***	0.047	0.119	0.282	0.029	0.082	**-.315***	-0.232	0.137	0.273
	Sig. (2-tailed)	0.038	0.768	0.452	0.07	0.853	0.604	0.042	0.14	0.387	0.08
PgHDZ18-39	Correlation Coefficient	-0.168	-0.07	**-.311***	-0.161	-0.039	-0.125	0.139	-0.101	0.082	-0.087
	Sig. (2-tailed)	0.287	0.657	0.045	0.308	0.807	0.431	0.379	0.525	0.604	0.584
PgHDZ19-01	Correlation Coefficient	**-.336***	0.042	-0.181	-0.102	-0.174	-0.094	-0.059	0.022	-0.278	-0.198
	Sig. (2-tailed)	0.03	0.79	0.25	0.52	0.271	0.555	0.708	0.888	0.075	0.209
PgHDZ19-02	Correlation Coefficient	0.29	0.123	0.235	**.352***	0.098	-0.07	-0.02	-0.226	**.393***	**.348***
	Sig. (2-tailed)	0.063	0.438	0.133	0.022	0.535	0.661	0.9	0.151	0.01	0.024
PgHDZ19-03	Correlation Coefficient	-0.209	0.286	-0.166	**.392***	0.088	-0.108	0.178	0.073	0.029	0.156
	Sig. (2-tailed)	0.184	0.066	0.293	0.01	0.578	0.497	0.26	0.647	0.855	0.325
PgHDZ19-06	Correlation Coefficient	**-.330***	0.021	-0.129	-0.178	-0.037	-0.245	-0.064	0.172	**-.395****	**-.322***
	Sig. (2-tailed)	0.033	0.893	0.415	0.259	0.814	0.119	0.689	0.275	0.01	0.038
PgHDZ19-07	Correlation Coefficient	0.184	-0.138	**-.317***	0.005	**-.329***	-0.079	-0.043	-0.131	0.078	-0.085
	Sig. (2-tailed)	0.244	0.383	0.041	0.974	0.033	0.62	0.788	0.407	0.622	0.592
PgHDZ22-01	Correlation Coefficient	-0.213	**.367***	-0.25	0.166	-0.087	-0.005	-0.111	-0.141	-0.024	0.038
	Sig. (2-tailed)	0.176	0.017	0.11	0.293	0.582	0.976	0.484	0.372	0.879	0.811
PgHDZ22-03	Correlation Coefficient	0.19	0.283	-0.025	**.349***	0.054	-0.037	0.187	0.192	0.064	0.228
	Sig. (2-tailed)	0.228	0.069	0.876	0.024	0.733	0.815	0.235	0.223	0.686	0.146
PgHDZ22-05	Correlation Coefficient	0.053	0.113	0.088	0.121	**.366***	0.064	-0.009	0.141	-0.003	0.061
	Sig. (2-tailed)	0.74	0.476	0.579	0.444	0.017	0.685	0.953	0.374	0.983	0.699
PgHDZ22-06	Correlation Coefficient	0.128	**.344***	0.072	0.077	0.192	0.066	-0.123	-0.061	0.079	0.099
	Sig. (2-tailed)	0.42	0.026	0.648	0.626	0.222	0.678	0.439	0.701	0.621	0.533
PgHDZ23-01	Correlation Coefficient	-0.258	-0.082	**-.384***	-0.223	-0.082	**-.318***	-0.271	-0.042	-0.154	**-.340***
	Sig. (2-tailed)	0.099	0.604	0.012	0.156	0.607	0.04	0.083	0.79	0.331	0.028
PgHDZ25-01	Correlation Coefficient	**-.368***	0.005	-0.074	-0.102	-0.002	0.064	-0.19	0.155	-0.204	-0.187
	Sig. (2-tailed)	0.016	0.976	0.643	0.52	0.989	0.689	0.229	0.326	0.195	0.235
PgHDZ26-02	Correlation Coefficient	-0.213	0.028	-0.196	-0.041	-0.158	**-.407****	0.001	-0.074	**-.399****	-0.29
	Sig. (2-tailed)	0.175	0.861	0.214	0.798	0.316	0.008	0.998	0.642	0.009	0.062
PgHDZ26-03	Correlation Coefficient	-0.292	0.221	**-.310***	-0.035	-0.083	-0.261	0.031	-0.099	-0.002	-0.157
	Sig. (2-tailed)	0.06	0.16	0.046	0.826	0.602	0.095	0.845	0.533	0.991	0.322
PgHDZ27-04	Correlation Coefficient	0.245	0.295	0.255	**.339***	0.284	0.013	0.205	0.043	0.285	**.348***
	Sig. (2-tailed)	0.118	0.058	0.103	0.028	0.069	0.935	0.193	0.785	0.067	0.024
