# Supplementary material for: Functional Study of PgHDZ01 Gene Involved in the Regulation of Ginsenoside Biosynthesis in Panax ginseng
Source: Plants (Basel). 2025 Nov 21;14(23):3562. doi: 10.3390/plants14233562 (PMC12693816; doi:10.3390/plants14233562)
Supplement: Supplementary file 1 [file plants-14-03562-s001.zip › Supplemental Table S2.pdf]

Supplemental Table S2. The correlation analysis of *HD-Zip* genes SNP mutation and ginsenoside content.SNP markers in 42 genotypes of cultivars were scored as three cases: “0” = reference allele homozygote, “1” = SNP mutant heterozygote, and “2” = SNP mutant homozygote. If two cases (0, 1 or 0, 2 or 1, 2) were present in 42 cultivars, the two-tailed student’s t-test was performed to determine the association between SNP and ginsenoside contents. If three cases (0, 1, 2) were all present in 42 cultivars, the association analysis between SNP and ginsenoside contents was conducted one-way analysis of variance (ANOVA) with post-hoc comparisons using the LSD test.

**Gene**	**SNP Position**	**Effect % (*P*-value)**								
		**Rg1**	**Rf**	**Rb1**	**Rg2**	**Rc**	**Rb2**	**Rb3**	**Rd**	**TS**
PgHDZ01	1300			57.0 (6.26E-04) (0/1)					42.0 (5.12E-03) (0/1)	32.7 (1.18E-03) (0/1)
PgHDZ09	359			48.1 (3.28E-02) (0/2)		88.0 (1.52E-04) (0/2)		142.8 (4.31E-03) (0/2)<br>72.9 (2.05E-02) (1/2)		31.6 (1.50E-02) (0/2)
	1316					80.7 (8.69E-04) (0/2)<br>36.5(1.78E-02) (1/2)		140.3 (4.78E-03) (0/2)<br>84.8E (1.09E-02) (1/2)		
PgHDZ11	399	42.0 (8.96E-03) (0/2)								
	473	35.5 (7.56E-03) (0/2)<br>32.4 (5.45E-03) (1/2)			229.8 (3.47E-02) (0/2)<br>225.8 (1.99E-02) (1/2)					
	577					46.2 (1.85E-02) (0/2)<br>56.6 (3.96E-03) (1/2)				
	902					46.2 (1.85E-02) (0/2)<br>56.6 (3.96E-03) (1/2)				
PgHDZ12-03	1340	60.0 (2.08E-02) (0/1)	89.3 (4.02E-02) (1/2)			59.2 (6.90E-04) (0/2)<br>80.2 (7.67E-04) (1/2)				
	1389	60.0 (2.08E-02) (0/1)	89.3 (4.02E-02) (1/2)			59.2 (6.90E-04) (0/2)<br>80.2 (7.67E-04) (1/2)				
PgHDZ13-07	529						61.2 (3.03E-03) (0/1)		56.3 (4.73E-02) (0/2)	
	578						71.9 (1.45E-03) (0/1)<br>87.9 (2.34E-03) (1/2)			
	579						80.6 (1.30E-03) (0/1)<br>50.2 (3.66E-02) (1/2)			
PgHDZ17-05	684			32.5 (3.84E-02) (0/1)<br>52.8 (1.56E-02) (0/2)	56.6 (4.57E-02) (0/1)<br>79.6 (4.00E-02) (0/2)	51.3 (3.38E-02) (0/2)	34.6 (1.13E-02) (0/1)<br>58.7 (2.73E-03) (0/2)	116.9 (6.65E-03) (0/1)	53.1 (1.27E-02) (0/2)	21.8 (1.57E-02) (0/1)<br>41.2 (1.40E-03) (0/2)
