# Supplementary material for: Functional Study of PgHDZ01 Gene Involved in the Regulation of Ginsenoside Biosynthesis in Panax ginseng
Source: Plants (Basel). 2025 Nov 21;14(23):3562. doi: 10.3390/plants14233562 (PMC12693816; doi:10.3390/plants14233562)
Supplement: Supplementary file 1 [file plants-14-03562-s001.zip › Supplemental Table S3.pdf]

Supplemental Table S3. The primers of gene clone.

Gene	Primer
PgHDZ01-VIGS	F- CGGAATTCCTATCCACCTCAAGAGAAGAAGAAC
	R- GGGGTACCGCTATCCGATAACCCTTTCTCTAC
PgHDZ01	F- TCCCCCCGGGATGCAGCGGTTCAGTTCAACAAAT
	R- TCCCCCCGGGTTACCAATGAAGGTTTGGTGCTTGAT
pTRV2-VIGS	F- GCTTTCCACAAATCTCTAACTTCGT
	R- TTGCCTTTGTAACCATCATCACTT
PgPDS-VIGS	F- CGGAATTCGACTGGCTTACACATTTTCTTTGG
	R- GGGGTACCGAAGAAATCGGTTCAAAGCAATC
