# Supplementary material for: Functional Study of PgHDZ01 Gene Involved in the Regulation of Ginsenoside Biosynthesis in Panax ginseng
Source: Plants (Basel). 2025 Nov 21;14(23):3562. doi: 10.3390/plants14233562 (PMC12693816; doi:10.3390/plants14233562)
Supplement: Supplementary file 1 [file plants-14-03562-s001.zip › Supplemental Table S4.pdf]

Supplemental Table S4. The qRT-PCR primer of genes.

Gene（NCBI accession number）	Primer
PgFPS (DQ087959.1)	F-GGATGATTATCTGGATTGCTTTGG
	R-CAGTGCTTTTACTACCAACCAGGAG
PgDDS (AB122080.1)	F- CGGAACGATTGACACTATTCTGAC
	R- CTGACCCAATCATCGTGCTGT
UGT71A27 (*UGTPg1,* KM491309.1)	F- TGCGTCCGTCTATCCCTAAAG
	R- TGATGTCCTGTCCAAGAATCCTAC
CYP716A47 (JN604536.1)	F- TTAGGTGATACGGCGGCAG
	R- CTGGGGGATGCGTTTTGTAT
PgHDZ01-Q	F- TGCCGAATGGTTTAGACGAC
	R- CCAGTGTTACTTTTTTCCCAGG
PgPDS-Q	F: GTGGACAGGCGTATGTTGAG
	R: CTGGGTTGATGAAGTTTAATGAC
