# Supplementary material for: Functional Study of PgHDZ01 Gene Involved in the Regulation of Ginsenoside Biosynthesis in Panax ginseng
Source: Plants (Basel). 2025 Nov 21;14(23):3562. doi: 10.3390/plants14233562 (PMC12693816; doi:10.3390/plants14233562)
Supplement: Supplementary file 1 [file plants-14-03562-s001.zip › Supplemental Table S5.pdf]

Supplemental Table S5. The primers for positive material detection.

Number	Fragment Position	Primer	Fragment length(bp)
a	Target gene	*PgHDZ01* : F- TCCCCCCGGGATGCAGCGGTTCAGTTCAACAAAT	819
		PgHDZ01 : R- TCCCCCCGGGTTACCAATGAAGGTTTGGTGCTTGAT	
b	Vector sequence containing the target gene	3301-F: CGCTCTTTCTTTCCAAGGTAATAG	1838
		3301-R: GTTGTACTCCAT CTTATTGCCCAG	
c	Target gene + partial sequence of upstream vector	3301-F: CGCTCTTTCTTTCCAAGGTAATAG	1397
		PgHDZ01 : R- TCCCCCCGGGTTACCAATGAAGGTTTGGTGCTTGAT	
d	Target gene + partial sequence of downstream vector	*PgHDZ01* : F- TCCCCCCGGGATGCAGCGGTTCAGTTCAACAAAT	1227
		3301-R: GTTGTACTCCAT CTTATTGCCCAG	
e	*RolC* gene	*RolC* -F: ATGGCTGAAGACGACTTGT GTTC	586
		RolC -R: TTAGCCGATTGCAAACTT	
